# Photocatalytic Enhancement and Recyclability in Visible-Light-Responsive 2D/2D g-C_3_N_4_/BiOI p-n Heterojunctions via a Z-Scheme Charge Transfer Mechanism

**DOI:** 10.3390/molecules29225418

**Published:** 2024-11-17

**Authors:** Shuo Yang, Tianna Wu, Kaiyue Li, Ping Huang, Wenhui Li, Yuquan Zhuo, Keyan Liu, Ziwen Yang, Donglai Han

**Affiliations:** 1 School of Materials Science and Engineering, Changchun University, Changchun 130022, China; wershi98@163.com (T.W.); lkaiyue29@163.com (K.L.); huangping_2023@163.com (P.H.); 19810911969@163.com (W.L.); zyq19942867075@163.com (Y.Z.); 2 Laboratory of Materials Design and Quantum Simulation College of Science, Changchun University, Changchun 130022, China; 3 School of Materials Science and Engineering, Changchun University of Science and Technology, Changchun 130022, China; liukeyan0910@163.com (K.L.); yzw123456789yzw@163.com (Z.Y.)

**Keywords:** photocatalysis, g-C_3_N_4_/BiOI p-n heterojunction, efficient and recyclable, Z-scheme mechanism

## Abstract

With the intensification of the energy crisis and the growing concern over environmental pollution, particularly the discharge of organic dye pollutants in industrial wastewater, photocatalytic degradation of these contaminants using solar energy has emerged as an effective, eco-friendly solution. In this study, we successfully synthesized 2D/2D g-C_3_N_4_/BiOI p-n heterojunctions via a simple precipitation method and a high-temperature calcination method. The unique 2D structures of g-C_3_N_4_ nanosheets (NSs) and BiOI NSs, coupled with the synergistic effect between the two materials, significantly enhanced the photocatalytic degradation performance of the heterojunctions under simulated sunlight. The band structures, as determined by Tauc curves, Mott–Schottky curves and XPS-VB analysis, revealed a Z-scheme charge transfer mechanism that efficiently reduced charge carrier recombination and improved electron–hole separation. The photocatalytic activity of 2D/2D g-C_3_N_4_/BiOI p-n heterojunctions for rhodamine B (Rh B) degradation reached 99.7% efficiency within 60 min, a 2.37-fold and 1.27-fold improvement over pristine BiOI NSs and g-C_3_N_4_ NSs, respectively. Furthermore, the heterojunction exhibited excellent recyclability stability, with the degradation efficiency decreasing by only 1.2% after five cycles. Radical scavenging experiments confirmed the involvement of superoxide radicals (∙O_2_^−^) and hydroxyl radicals (∙OH) as the primary reactive species in the degradation process. This work highlights the potential of 2D/2D g-C_3_N_4_/BiOI p-n heterojunctions for efficient photocatalytic applications in environmental remediation.

## 1. Introduction

With the intensification of the energy crisis and worsening environmental pollution, particularly the massive discharge of organic dye pollutants in industrial wastewater, the demand for green and renewable energy solutions has become increasingly urgent [1,2]. Photocatalytic degradation of organic pollutants in water using solar energy by semiconductor materials has emerged as an efficient, eco-friendly, and cost-effective approach to address these challenges, offering a promising alternative to traditional water treatment technologies [3,4]. In recent years, substantial efforts have been devoted to enhancing the efficiency, long-term stability, and cost-effectiveness of semiconductor photocatalysts [5]. Simultaneously, numerous semiconductor materials with visible light response characteristics have been discovered, significantly expanding the possibilities for practical photocatalytic applications [6]. Among the many visible-light-responsive semiconductors, g-C_3_N_4_, a two-dimensional (2D) sp^2^-conjugated with carbon atoms arranged in a honeycomb lattice and a suitable band gap of 2.7 eV, has garnered increasing attention due to its easy preparation, environmental friendliness, and good thermal and chemical stability [7,8], making it a promising candidate for environmental pollution control. However, its photogenerated carrier recombination rate limits its photocatalytic efficiency, hindering its practical applications [9].

Various strategies, such as morphology control, element doping and heterojunction construction, have been explored to optimize the photocatalytic performance of g-C_3_N_4_ [10]. Among these methods, the construction of heterojunctions [11] is the most widely used in recent years. So far, many semiconductor materials have been reported to form heterojunction structures with g-C_3_N_4_, including Co_3_O_4_ [12], CuO [13], ZnO [14], Bi_X_O_Y_I_Z_ [15], Fe_2_O_3_ [16], and Ag_3_VO_4_ [17]. The construction of a 2D/2D heterostructure photocatalyst is widely regarded as an effective strategy for achieving efficient separation of photoinduced electron–hole pairs, thereby enhancing photocatalytic activity [18,19]. Zhang et al. [11] developed 2D/2D BiOBr/g-C_3_N_4_ heterojunctions via an in situ self-assembly method, achieving a 99% degradation efficiency of Rh B (10 mg·L^−1^) under simulated sunlight in only 30 min, while demonstrating excellent stability. Wang et al. [20] synthesized an ultrathin g-C_3_N_4_/Bi_2_WO_6_ composite using a bottom-up approach, achieving an impressive IBF degradation efficiency of 96% with a 25% g-C_3_N_4_/Bi_2_WO_6_ composition. BiOI, with a band gap of approximately 1.8 eV, is a good visible-light-responsive semiconductor photocatalyst featuring a unique 2D layered structure [21]. This arrangement structure can generate an internal electrostatic field, which facilitates the migration and separation of photogenerated electron–hole pairs and retains electron and hole pairs with strong redox abilities [11,14,22]. Moreover, the energy levels of BiOI can be well matched with those of g-C_3_N_4_, making it an ideal candidate for constructing heterojunctions. Therefore, the assembly of a 2D/2D heterojunction between 2D g-C_3_N_4_ and 2D BiOI could provide additional charge transfer pathways, reduce photogenerated carrier recombination and enhance carrier separation, thereby improving photocatalytic performance [23].

g-C_3_N_4_ can be synthesized through a variety of methods, including thermal decomposition [24,25], hydrothermal synthesis [26,27], template-assisted approaches [28], solvothermal synthesis, microwave-assisted techniques, and chemical vapor deposition. Similarly, BiOI can be prepared using methods such as hydrothermal and solvothermal synthesis, sol–gel processes, electrochemical deposition, template techniques, and microwave-assisted methods. Among these, the synthesis of g-C_3_N_4_ via thermal decomposition and of BiOI via the precipitation method, followed by calcinating the mix of them to form a 2D/2D g-C_3_N_4_/BiOI heterojunction, stands out for its simplicity, ease of implementation and cost-effectiveness, making it particularly suitable for large-scale photocatalyst production. The resulting 2D/2D g-C_3_N_4_/BiOI heterojunctions demonstrate significantly enhanced photocatalytic performance due to the synergistic interaction between the two materials, which effectively reduces charge carrier recombination and improves overall efficiency.

In this study, 2D/2D g-C_3_N_4_/BiOI p-n heterojunctions with a Z-scheme charge transfer mechanism were successfully synthesized via a precipitation method combined with a high-temperature calcination method. The synergistic effect between the two sheet-structured BiOI NSs and the g-C_3_N_4_ NSs significantly improves the separation of photogenerated carriers in heterojunctions, leading to a marked improvement in photocatalytic performance. The photocatalytic activities of BiOI NSs, g-C_3_N_4_ NSs, and 2D/2D g-C_3_N_4_/BiOI p-n heterojunctions were evaluated by the photocatalytic degradation of rhodamine B (Rh B) under simulated sunlight irradiation. This study focuses on the structural characteristics, recyclability stability and photocatalytic mechanism of g-C_3_N_4_/BiOI p-n heterojunctions, as well as the impact of the BiOI to g-C_3_N_4_ ratio on enhancing photocatalytic activity.

## 2. Results and Discussion

### 2.1. Structure and Morphology of BiOI, g-C_3_N_4_ and g-C_3_N_4_/BiOI

**Figure 1:**
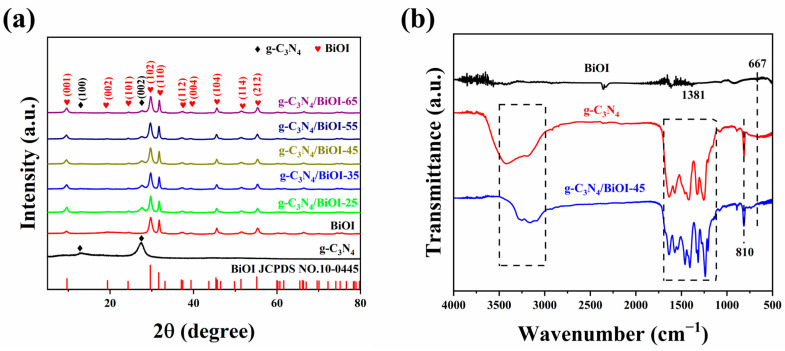
XRD patterns (**a**) and FT-IR spectra (**b**) of BiOI NSs, g-C_3_N_4_ NSs, and g-C_3_N_4_/BiOI heterojunctions.

The structures and chemical bonds of BiOI NSs, g-C_3_N_4_ NSs, and g-C_3_N_4_/BiOI heterojunctions were characterized using XRD and FT-IR. Figure 1a displays the XRD patterns of the prepared BiOI NSs, g-C_3_N_4_ NSs, and g-C_3_N_4_/BiOI-(25-65) heterojunctions. Two distinct peaks of g-C_3_N_4_ NSs appear at approximately 13.0° and 27.5°, which align with the (100) and (002) lattice planes of g-C_3_N_4_ [29] (JCPDS Card No. 87-1526). The major diffraction peaks of BiOI NSs are located at around 9.7°, 29.7°, 31.7°, 45.4°, and 55.2°, corresponding to the (001), (102), (110), (200), and (212) crystal planes of the tetragonal phase BiOI [30] (JCPDS Card No. 10-0445). In the XRD image of the g-C_3_N_4_/BiOI heterojunctions, characteristic diffraction peaks of both tetragonal BiOI NSs and g-C_3_N_4_ NSs are present without any observable impurity peaks, indicating the successful synthesis of the nanocomposites. These pronounced diffraction peaks reflect the crystalline nature of the samples, allowing for an estimation of the average crystallite size using the Scherrer equation: D= 0.9λ/βcos⁡θ,where λ is the wavelength of the X-ray radiation, β represents the full width at half maximum of the peak, and θ is the diffraction angle. The average crystallite size of samples g-C_3_N_4_/BiOI-(25-65) remained nearly unchanged as the amount of BiOI NSs increased in g-C_3_N_4_/BiOI heterojunctions, suggesting that nitrogen is not incorporated into the BiOI bulk phase but exists as a bismuth oxyhalide [31].

Figure 1b presents the FT-IR spectra of the prepared BiOI NSs, g-C_3_N_4_ NSs, and g-C_3_N_4_/BiOI-45 heterojunctions. The FT-IR spectrum of BiOI NSs exhibits absorption peaks at 667 cm^−1^ and 1381 cm^−1^, in accordance with the bending vibration of Bi-O bonds [32,33,34] and stretching vibration of I-O bonds [35,36], respectively. For g-C_3_N_4_ NSs, a distinct absorption peak at 810 cm^−1^ is observed, corresponding to the out-of-plane bending vibration of its heptazine ring [37,38], while peaks occurring within 1700–1200 cm^−1^ are attributed to the skeletal stretching vibrations of C-N heterocycles [39]. Additionally, broad peaks between 3500 and 3000 cm^−1^ are ascribed to the stretching vibrations of -N-H and -O-H bonds [40]. The FT-IR spectrum of the g-C_3_N_4_/BiOI-45 heterojunction shows no additional peaks beyond those associated with g-C_3_N_4_ NSs and BiOI NSs, confirming the successful preparation of the heterojunction. The absence of new peaks indicates that the two materials were combined through simple physical mixing rather than chemical bonding. However, due to the stronger intensity of the g-C_3_N_4_ peaks, the Bi-O bond absorption peak at 667 cm^−1^ is difficult to distinguish, necessitating further analysis via XPS, EDS, and HRTEM.

**Figure 2:**
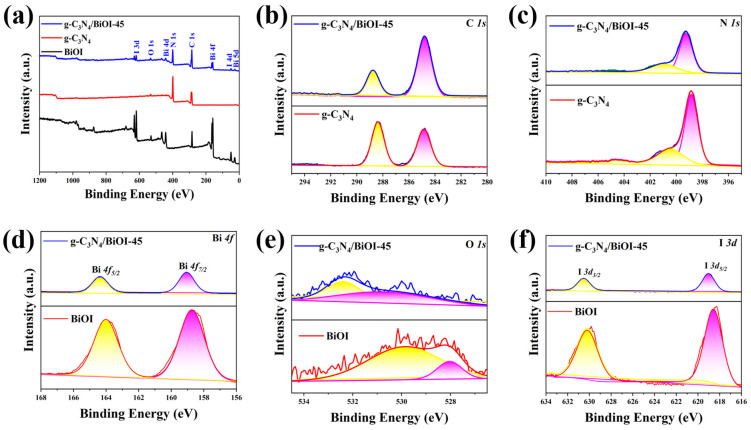
Full spectra (**a**); high-resolution XPS spectra of C *1s*, N *1s*, Bi *4f*, O *1s*, and I *3d* (**b**–**f**).

To conduct a deeper analysis of the elemental composition and chemical state of the synthesized nanocomposites, XPS was utilized. All the binding energy values are corrected by the C *1s* peak value (284.8 eV) of the indefinite carbon [41]. Figure 2a shows the XPS survey spectra of BiOI NSs, g-C_3_N_4_ NSs and g-C_3_N_4_/BiOI-45 heterojunction, confirming the presence of Bi, O, and I elements on the surface of BiOI NSs; C and N elements on the surface of g-C_3_N_4_ NSs; and C, N, Bi, O, and I elements on the surface of g-C_3_N_4_/BiOI-45 heterojunction. The observed XPS survey spectra display characteristic peaks corresponding to these elements, with no detectable impurities. Figure 2b,c present the high-resolution XPS spectra of C and N elements in g-C_3_N_4_ NSs and the g-C_3_N_4_/BiOI-45 heterojunction, while Figure 2d–f shows the high-resolution XPS spectra of Bi, O, and I elements in BiOI NSs and the g-C_3_N_4_/BiOI-45 heterojunction. From Figure 2b, we can see that the C *1s* spectrum of g-C_3_N_4_/BiOI-45 exhibits two peaks, located at 284.8 eV and 288.7 eV, where the peak at 284.8 eV is attributed to adventitious carbon used for calibration [42], and the peak at 288.7 eV corresponds to *sp^2^*-bonded carbon in the triazine rings (N-C=N) within the aromatic structure of g-C_3_N_4_ [43]. In Figure 2c, the N *1s* peaks in g-C_3_N_4_/BiOI-45 are observed at 401.2 eV and 399.4 eV, respectively, corresponding to N-H and C-N bonds [41]. As shown in Figure 2d, the two peaks of the Bi *4f* spectrum are located at 159.0 eV and 164.3 eV, corresponding to the Bi^3+^ *4f*_7/2_ and *4f*_5/2_ peaks in BiOI [44,45,46]. In Figure 2e, the two peaks of the O 1s spectrum at 532.3 eV and 530.2 eV are consistent with oxygen vacancies and Bi-O bonds [47]. The two peaks in the I *3d* spectrum of g-C_3_N_4_/BiOI at 630.4 eV and 619.0 eV correspond to the I *3d_5/2_* and I *3d_3/2_* orbitals [48,49] (Figure 2f). In summary, compared to g-C_3_N_4_ NSs and BiOI NSs, the binding energies of C *1s*, N *1s*, Bi *4f*, O *1s*, and I *3d* in the g-C_3_N_4_/BiOI-45 heterojunction have shifted, confirming a successful composite reaction in the conjunction of g-C_3_N_4_ and BiOI. Like the C *1s* peak, the N *1s* peak in the g-C_3_N_4_/BiOI-45 heterojunction shifts to a higher binding energy, which can further confirm the occurrence of electron transfer between the two components.

**Figure 3:**
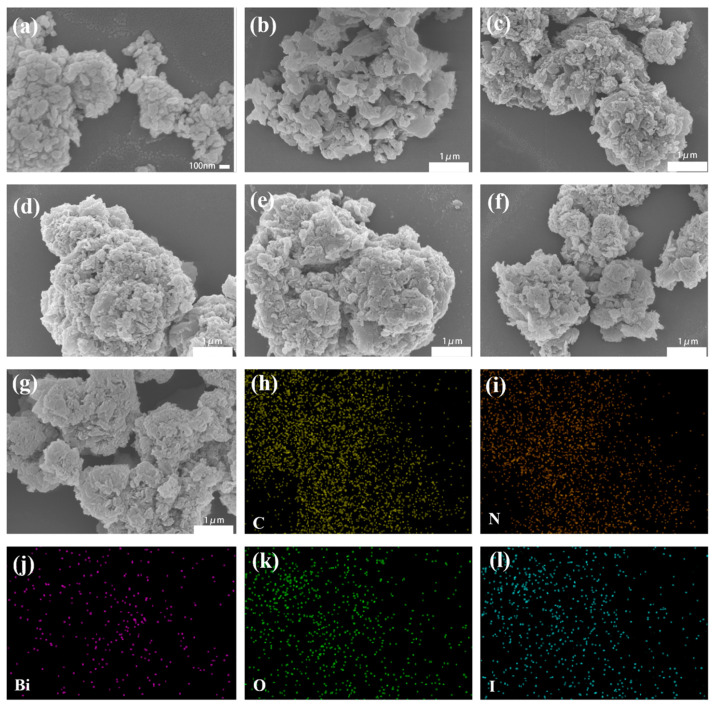
SEM image of BiOI NSs (**a**), g-C_3_N_4_ NSs (**b**), and g-C_3_N_4_/BiOI-(25-65) heterojunctions (**c**–**g**); EDS images of g-C_3_N_4_/BiOI-45 heterojunctions (**h**–**l**).

The prepared samples were subjected to SEM to reveal their morphology. As shown in Figure 3a, BiOI exhibits an irregular nanosheet morphology with diameters ranging from approximately 40 to 110 nm. Figure 3b reveals that g-C_3_N_4_ displays a smooth nanosheet with an average size of about 200–800 nm. Furthermore, Figure 3c–g displays SEM images of g-C_3_N_4_/BiOI nanocomposites with different mass ratios. As shown in the images, the surface of the composite becomes rougher, indicating that BiOI NSs have been successfully deposited onto the g-C_3_N_4_ NSs. Additionally, with an increase in the amount of deposited BiOI, the smaller BiOI NSs tend to aggregate into larger clusters. As depicted in Figure 3h–l, the C, N, Bi, O, and I elements are uniformly distributed in the g-C_3_N_4_/BiOI-45 samples, a finding that aligns with the XPS results.

**Figure 4:**
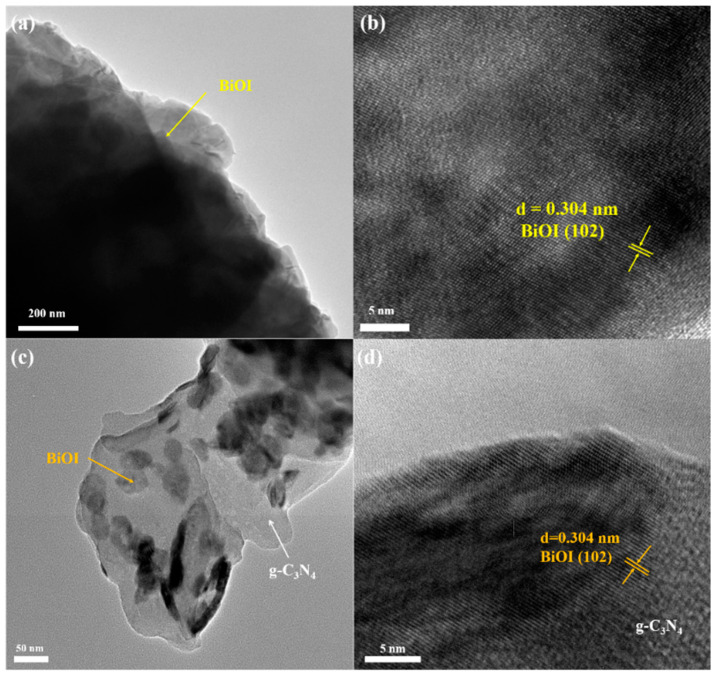
TEM (**a**) and HRTEM (**b**) images of BiOI NSs; TEM (**c**) and HRTEM (**d**) images of g-C_3_N_4_/BiOI-45 heterojunctions.

TEM and HRTEM analyses were employed to gain insights into the microstructure of BiOI NSs (Figure 4a,b) and the g-C_3_N_4_/BiOI-45 heterojunction (Figure 4c,d). Figure 4a clearly displays the layered nanosheet structure of BiOI, with multiple thin layers stacked together. The HRTEM image in Figure 4b reveals an interplanar spacing of approximately 0.304 nm, conforming to the lattice spacing of the tetragonal phase BiOI (102) plane [50]. Figure 4c illustrates a well-defined TEM image of g-C_3_N_4_/BiOI-45 heterojunctions, demonstrating the deposition of BiOI NSs on the surface of g-C_3_N_4_ NSs. In the HRTEM image (Figure 4d), a lattice spacing of 0.304 nm, corresponding to the (102) plane of tetragonal BiOI [50], is evident alongside the g-C_3_N_4_ structure. These findings are in accordance with the XRD results, confirming the successful integration of BiOI NSs and g-C_3_N_4_ NSs.

**Figure 5:**
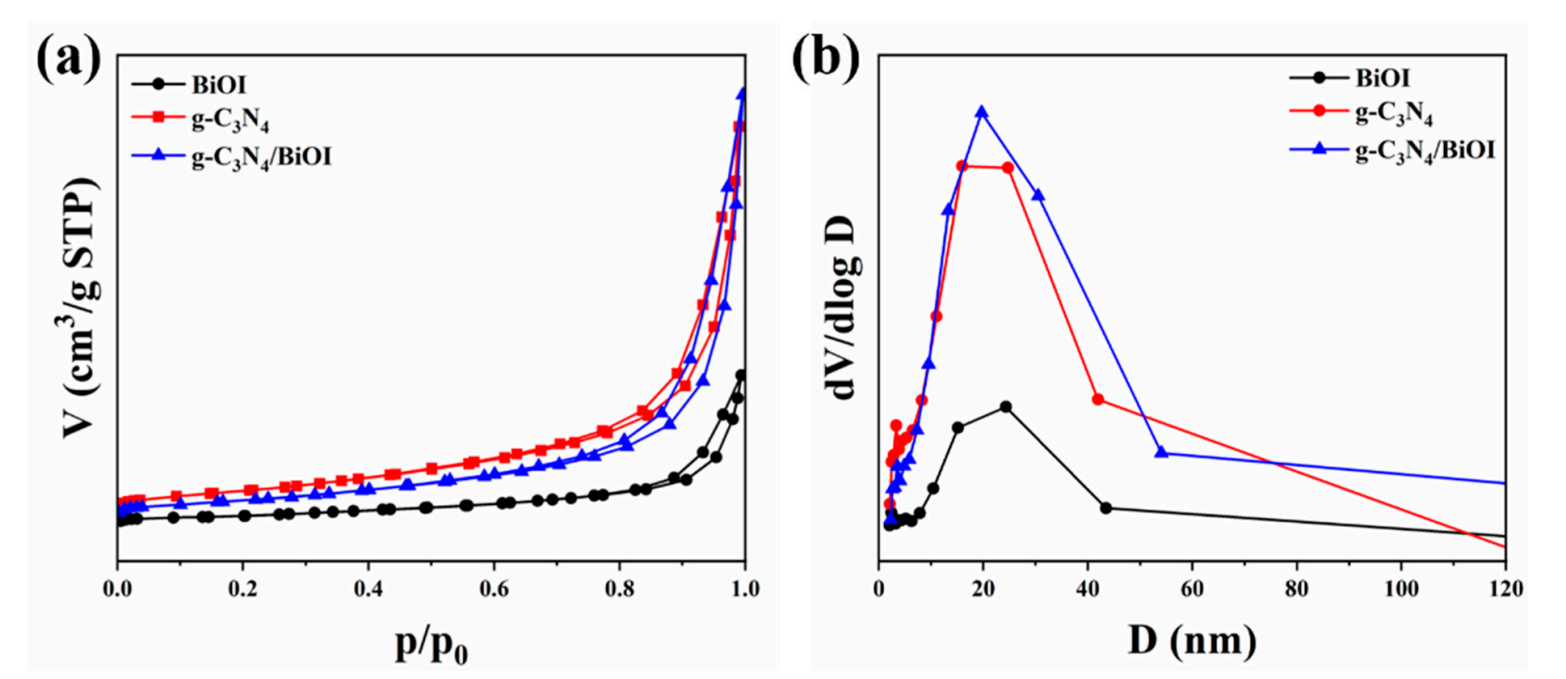
N_2_ adsorption–desorption isotherms (**a**) and pore diameter distributions (**b**) of BiOI NSs, g-C_3_N_4_ NSs, and g-C_3_N_4_/BiOI-45 heterojunctions.

The specific surface area and average pore diameter of BiOI NSs, g-C_3_N_4_ NSs, and g-C_3_N_4_/BiOI heterojunctions were investigated through N_2_ adsorption–desorption experiments, as shown in Figure 5. In Figure 5a, it can be observed that the BiOI, g-C_3_N_4_, and g-C_3_N_4_/BiOI samples all exhibit Type IV adsorption isotherms with H_3_ hysteresis loops. The specific surface areas of BiOI and g-C_3_N_4_ are 24.46 m^2^/g and 54.51 m^2^/g, respectively, both lower than that of g-C_3_N_4_/BiOI, which reaches 63.01 m^2^/g. As can be seen from Figure 5b, the pore size distribution of all samples is between 10 and 30 nm, indicating their mesoporous properties. Specifically, the average pore sizes of BiOI and g-C_3_N_4_ are 23.52 nm and 15.93 nm, respectively, both lower than that of g-C_3_N_4_/BiOI heterojunctions, which have an average pore size of 24.83 nm. In summary, the g-C_3_N_4_/BiOI heterojunctions demonstrate increased specific surface area and mesoporous architecture, which facilitates the acquisition of additional reactive sites, thereby enhancing the photocatalytic effectiveness [51] of g-C_3_N_4_/BiOI heterojunctions.

### 2.2. Mechanism Analysis

**Figure 6:**
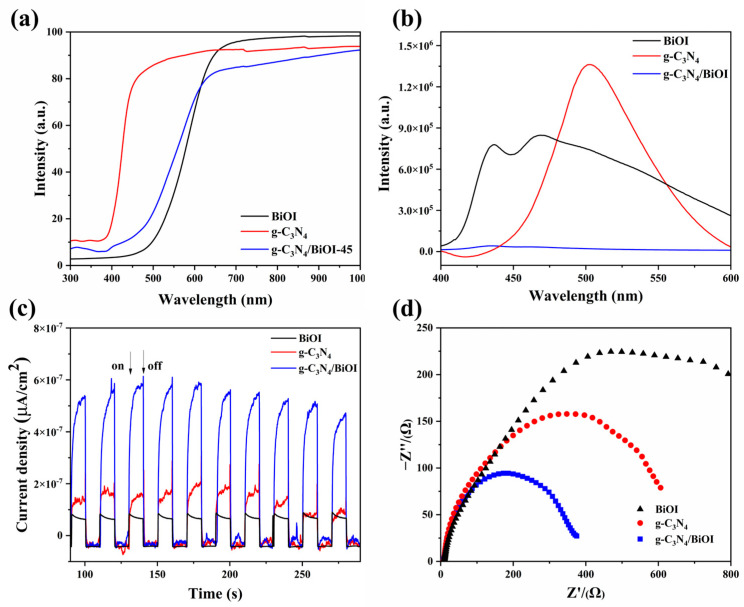
UV-Vis DRS (**a**), PL spectra (**b**), PC responses (**c**), and EIS Nyquist plots (**d**) of BiOI NSs, g-C_3_N_4_ NSs, and the g-C_3_N_4_/BiOI-45 heterojunction.

Figure 6a shows the UV-Vis diffuse reflectance spectra (DRS) of BiOI NSs, g-C_3_N_4_ NSs, and g-C_3_N_4_/BiOI-45 heterojunctions. The absorption edge of g-C_3_N_4_ NSs is at approximately 380 nm, while that of BiOI NSs is around 510 nm. The absorption edge of the g-C_3_N_4_/BiOI-45 heterojunction lies between those of g-C_3_N_4_ NSs and BiOI NSs, confirming the successful combination of the two materials. The electron–hole combination, separation, and transfer in the g-C_3_N_4_/BiOI-45 photocatalyst were investigated using photoluminescence (PL), photocurrent (PC) responses, and electrochemical impedance spectroscopy (EIS). As shown in Figure 6b, the PL intensity of the g-C_3_N_4_/BiOI-45 heterojunction is significantly lower than that of BiOI NSs and g-C_3_N_4_ NSs, indicating a pronounced suppression of the reassembly of photogenerated carriers after the integration of g-C_3_N_4_ and BiOI [52,53]. As shown in Figure 6c, the photocurrent intensity of the g-C_3_N_4_/BiOI-45 heterojunction is significantly higher than that of BiOI NSs and g-C_3_N_4_ NSs, indicating that g-C_3_N_4_/BiOI-45 can produce more photogenerated carriers than pure BiOI and g-C_3_N_4_ [51]. In parallel, as depicted in Figure 6d, the semicircle of the g-C_3_N_4_/BiOI-45 heterojunction is much smaller than those of BiOI NSs and g-C_3_N_4_ NSs, signifying a lower charge transfer resistance in the g-C_3_N_4_/BiOI-45 sample [54,55]. These findings suggest that the synthesized g-C_3_N_4_/BiOI-45 heterojunction exhibits superior photoelectrical properties and more efficient electron–hole separation and transformation.

### 2.3. Photocatalytic Activity

**Figure 7:**
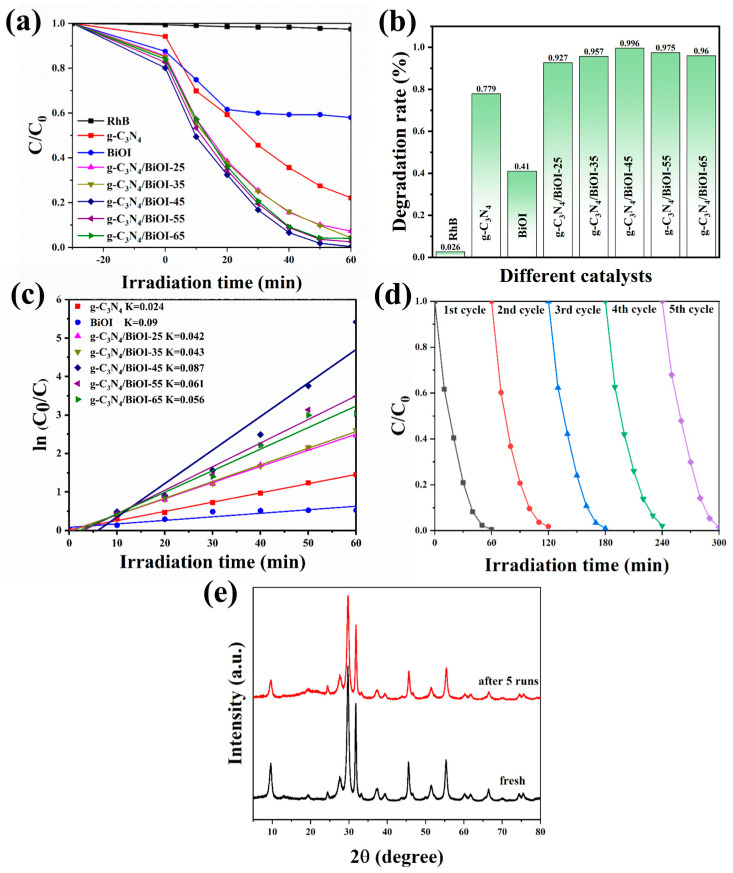
Photocatalytic degradation curves of Rh B under simulated sunlight irradiation (**a**) and a bar chart of the degradation rate at 60 min (**b**) for BiOI NSs, g-C_3_N_4_ NSs, and g-C_3_N4/BiOI heterojunctions; corresponding first-order kinetic curves (**c**); cycling runs of g-C_3_N_4_/BiOI-45 for degradation of Rh B (**d**); XRD patterns of g-C_3_N_4_/BiOI-45 before and after Rh B degradation cycles (**e**).

Under simulated sunlight, we assessed the photocatalytic operation of the prepared g-C_3_N_4_/BiOI heterojunctions by degrading Rh B. It can be clearly seen from Figure 7a,b that, in the absence of a photocatalyst, the degradation efficiency of Rh B under simulated sunlight is extremely low, at only 2.6%, which can be considered negligible. In contrast to the individual photocatalytic performances of g-C_3_N_4_ NSs and BiOI NSs, the g-C_3_N_4_/BiOI heterojunctions show a significant improvement in photocatalytic activity towards Rh B. Particularly, the g-C_3_N_4_/BiOI-45 heterojunction exhibits the best degradation efficiency, reaching 99.7% degradation within 60 min. Its photocatalytic efficiency exhibits an enhancement of 2.37-fold and 1.27-fold compared to pure BiOI NSs and g-C_3_N_4_ NSs, respectively. Figure 7c presents the quantitative analysis of the photocatalytic reaction kinetics using a first-order kinetic model to determine the overall reaction rate. The kinetics are described by the following equation [56]:

(1) $$\ln\left(C_{t}/C_{0}\right)=-Kt$$

where *K* represents the rate constant for degradation, *C*_0_ denotes the initial concentration of the pollutant, and *C_t_* signifies the concentration of the pollutant at time *t*. Under visible light irradiation, the photocatalytic degradation kinetics of Rh B were systematically evaluated for g-C_3_N_4_, BiOI, g-C_3_N_4_/BiOI-25, g-C_3_N_4_/BiOI-35, g-C_3_N_4_/BiOI-45, g-C_3_N_4_/BiOI-55, and g-C_3_N_4_/BiOI-65. The corresponding degradation rate constants were 0.024 min^−1^, 0.009 min^−1^, 0.042 min^−1^, 0.043 min^−1^, 0.087 min^−1^, 0.061 min^−1^, and 0.056 min^−1^, respectively. Notably, the g-C_3_N_4_/BiOI-45 heterojunction demonstrated the highest photocatalytic activity, with a degradation rate constant approximately 3.63 times that of pure g-C_3_N_4_ NSs and 9.67 times that of BiOI NSs. To perform additional analysis on the recyclability and stability of the g-C_3_N_4_/BiOI-45 heterojunction, five cycles of photocatalytic degradation measurements of Rh B were conducted (Figure 7d). With five cycles completed, the degradation efficiency decreased by only 1.2%, and the XRD pattern of the photocatalyst after cycling (Figure 7e) remained consistent with that before cycling, indicating excellent reusability and structural stability of the g-C_3_N_4_/BiOI-45 heterojunction.

### 2.4. Photocatalytic Mechanism

**Figure 8:**
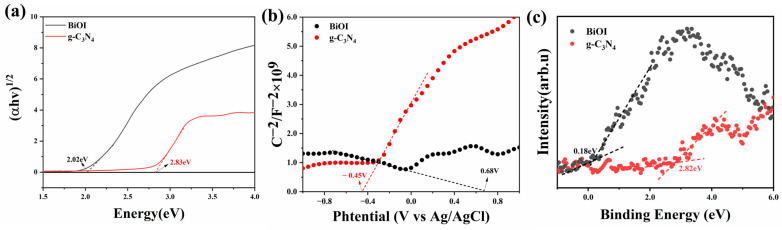
Tauc curves (**a**), Mott–Schottky curves (**b**), and VB-XPS spectra (**c**) of BiOI NSs and g-C_3_N_4_ NSs.

It is well established that effective band alignment is crucial for achieving high photocatalytic performance in heterojunction photocatalysts. To determine the band positions of the g-C_3_N_4_ NSs and BiOI NSs, Tauc plots, Mott–Schottky (M-S) curves, and XPS valence band (XPS-VB) spectra were employed to estimate the flat band potentials (Fermi energy level, *E_f_*), valence band maximum (VBM), and conduction band minimum (CBM). As shown in Figure 8a, the Tauc formula is given by:

(2) $$\alpha hv=K{\left(hv-E_{g}\right)}^{\frac{1}{2}}$$

where K represents the parameter related to material properties, h is the Planck constant, α signifies the absorption coefficient, and v is the frequency of the incident photons [57]. From Figure 8a, the bandgap energies ($E_{g}$) of BiOI NSs and g-C_3_N_4_ NSs were derived to be 2.02 eV and 2.83 eV, respectively. Based on the M-S curves, the *E_f_* of BiOI NSs and g-C_3_N_4_ NSs were found to be 0.68 eV and −0.45 eV (vs. Ag/AgCl), respectively. The positive slope of the M-S curve indicates that g-C_3_N_4_ is an n-type semiconductor, while the negative slope for BiOI confirms it as a p-type semiconductor. These values were then converted to the standard hydrogen electrode (NHE) potential using the following equation [58]:

(3) $$E_{NHE}=E_{Ag/AgCl}+0.198\;V$$

Therefore, the *E_f_* values of BiOI NSs and g-C_3_N_4_ NSs under NHE conditions were calculated as 0.88 V and −0.25 V, respectively. The relative potential values of VBM to *E_f_* for BiOI NSs and g-C_3_N_4_ NSs were estimated using VB-XPS spectra (Figure 8c) and found to be 0.18 eV and 2.82 eV, respectively. In accordance with the *E_f_* values and VB-XPS results, the $E_{VB}$ values of BiOI NSs and g-C_3_N_4_ NSs were evaluated to be 1.06 V and 2.57 V vs. NHE, respectively. Subsequently, utilizing the following formula [59]:

(4) $$E_{g}=E_{VB}-E_{CB}$$

The $E_{CB}$ values of BiOI NSs and g-C_3_N_4_ NSs were estimated to be −0.96 V and −0.26 V, respectively.

**Figure 9:**
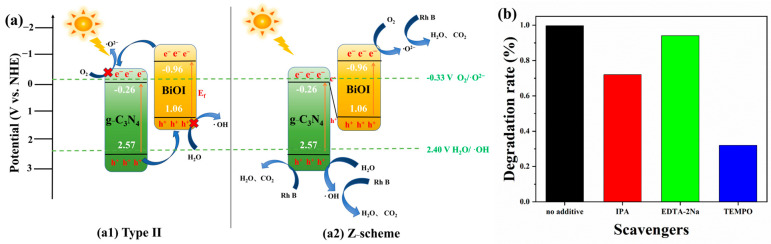
Schematic illustration of the photodegradation process (**a**) and radical scavenging experiments (**b**) for Rh B degradation under simulated sunlight using the g-C_3_N_4_/BiOI p-n heterojunctions.

Based on these band structure data, we plotted two possible mechanisms in the application of photocatalytic degradation of Rh B by g-C_3_N_4_/BiOI-45 p-n heterojunctions: the dual charge transfer mechanism and Z-scheme transfer mechanism. As illustrated in Figure 9(a1), under simulated sunlight irradiation, both BiOI NSs and g-C_3_N_4_ NSs are excited simultaneously, generating photogenerated electrons (e^−^) that transition from the VBM to the CBM, leaving holes (h^+^) in their respective VBMs. Subsequently, the e^−^ in the CBM of BiOI NSs migrates to the CBM of g-C_3_N_4_ NSs, while the h^+^ in the VBM of g-C_3_N_4_ NSs transfers to the VBM of BiOI NSs. This results in the e^−^ remaining in the CBM of g-C_3_N_4_ NSs with an energy level of −0.28 V, which is higher than the O_2_/∙O_2_^−^ potential (−0.33 V vs. NHE [41,48]). Consequently, the e^−^ in the CBM of g-C_3_N_4_ NSs cannot reduce O_2_ to ∙O_2_^−^. Similarly, h^+^ remain in the VBM of BiOI NSs with an energy level of 0.14 V, which is lower than the ∙OH/H_2_O potential (2.4 V vs. NHE [48]), preventing the h^+^ in the VBM of BiOI NSs from oxidizing H_2_O to produce ∙OH. Therefore, under this dual charge transfer mechanism, the only active species in the photocatalysts should be h^+^. In contrast, the potential double Z-scheme transfer mechanism, as depicted in Figure 9(a2), suggests that e^−^ in the CBM of g-C_3_N_4_ NSs recombine with h^+^ in the VBM of BiOI NSs, forming a built-in electric field at the interface, which prevents further recombination of electrons and holes. Subsequently, e^−^ remain in the CBM of BiOI NSs at an energy level of −1.88 V, which is lower than the O_2_/∙O_2_^−^ potential, allowing the electrons in the CBM of BiOI NSs to react with O_2_ to generate ∙O_2_^−^. Simultaneously, h^+^ remain in the VBM of g-C_3_N_4_ NSs with an energy level of 2.53 V, which is higher than the ∙OH/H_2_O potential, enabling the h^+^ in the VBM of g-C_3_N_4_ NSs to react with H_2_O to produce ∙OH. Under the Z-scheme electron transfer mechanism, the active species ∙O_2_^−^, ∙OH, and h⁺ can all react with pollutants, decomposing them into H_2_O and CO_2_.

To confirm the photocatalytic mechanism, radical scavenging experiments were performed. The procedure followed the same steps as the photocatalytic experiments, with the addition of specific scavengers (1 mmol/L) to the degradation solution. IPA, EDTA-2Na, and TEMPO were used to selectively capture hydroxyl radicals (∙OH), holes (h⁺), and superoxide radicals (∙O_2_^−^), respectively. As shown in Figure 9b, upon the addition of EDTA-2Na, the photocatalytic degradation efficiency of g-C_3_N_4_/BiOI-45 p-n heterojunctions for Rh B decreased from 99.7% to 94.1%, indicating the involvement of h⁺ as an active species in the photocatalytic process, albeit with a relatively minor impact. After the introduction of IPA, the degradation efficiency dropped to 72.0% suggesting that ∙OH also contributes to photocatalytic degradation. When TEMPO was added, the photocatalytic degradation efficiency further decreased to 32.0%, confirming that ∙O_2_^−^ plays the most significant role and is the primary active species. These results indicate that ∙O_2_^−^ and ∙OH are the dominant reactive intermediates, while h⁺ plays a less significant role in the degradation of Rh B. This observation aligns with our proposed Z-scheme transfer mechanism.

In recent years, researchers have made significant efforts in developing a wide range of photocatalysts, such as Bi_4_Ti_3_O_12_/AgI [60], Ag_2_CrO_4_/g-C_3_N_4_ [61], Bi_2_MoO_6_/Bi_2_Mo_3_O_12_ [62], BiVO_4_/g-C_3_N_4_ [63], Cu_2_O/BiOBr [64], etc., to enhance the degradation performance of Rh B. As shown in Table 1, it is evident that even when the components of the heterojunction photocatalysts are identical, the photocatalytic degradation efficiency varies due to different experimental conditions, such as the amount of catalyst used, the concentration of Rh B, the light source employed in photocatalytic degradation experiments, the distance between the sample and the light source, and the exposure time. Consequently, the degradation efficiency of Rh B also differs when different photocatalysts or experimental conditions are used.

## 3. Experiment

### 3.1. Experimental Materials

Pentahydrate bismuth nitrate (Bi(NO_3_)_3_·5H_2_O, AR, 99.0%) was purchased from Shanghai Macklin Biochemical Technology Co., Ltd., Shanghai, China, while potassium iodide (KI, AR, ≥99.0%), mannitol (C_6_H_14_O_6_, AR, 98.0%), urea (CO(NH_2_)_2_, ≥99.5%), rhodamine B (Rh B, AR), 4-hydroxy-TEMPO (TEMPO, 98%), Isopropyl alcohol (IPA, AR, ≥99.5%), and ethylenediaminetetraacetic acid disodium salt (EDTA-2Na, 0.1000 mol/L (0.1M)) were all acquired from Shanghai Aladdin Biochemical Technology Co., Ltd., Shanghai, China. All chemicals were employed without further purification.

### 3.2. Preparation of Heterojunction Materials

#### 3.2.1. Preparation of g-C_3_N_4_ NSs

To begin, we weighed a specific amount of CO(NH_2_)_2_ and placed it into a crucible, then heated it in a muffle furnace at a rate of 5 °C per minute until the temperature reached 550 °C; we subsequently calcined it for 2 h and then cooled it to room temperature. The resulting light-yellow solid was immersed in a nitric acid solution with a pH of 1 and stirred at 60 °C for 8 h. After washing several times with deionized water, the material was subjected to drying at 80 °C for 3 h. The dried product was then returned to the muffle furnace, where the temperature was raised at a rate of 5 °C per minute to 500 °C and calcined for another 2 h before cooling. This process yielded g-C_3_N_4_ NSs.

#### 3.2.2. Preparation of BiOI NSs

We dissolved 2.425 g of Bi(NO_3_)_3_·5H_2_O in 60 mL of deionized water and stirred it magnetically for 1 h. Simultaneously, we dissolved 5.0 mmol of KI in 40 mL of deionized water under magnetic stirring for 1 h. Subsequently, 0.25 g of C_6_H_14_O_6_ was added to the solution containing Bi(NO_3_)_3_·5H_2_O, and it continued to stir for 30 min. Then, the KI solution was slowly poured along the inner wall of the beaker into the Bi(NO_3_)_3_·5H_2_O solution. It was then stirred continuously at 60 °C for 3 h. The resulting BiOI nanomaterials were washed several times with deionized water and anhydrous ethanol, followed by vacuum drying at 60 °C for 12 h to obtain the BiOI NSs.

#### 3.2.3. Preparation of g-C_3_N_4_/BiOI heterojunctions

**Scheme 1:**
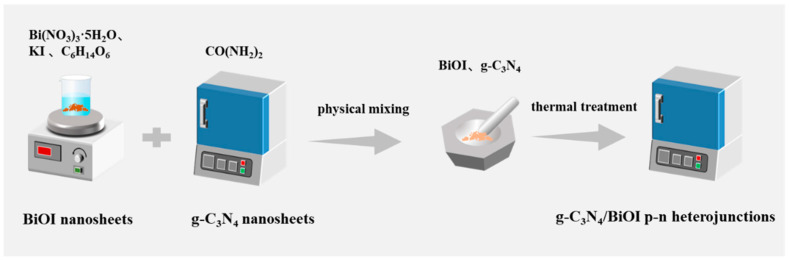
Synthesis process of g-C_3_N_4_/BiOI heterojunctions.

To prepare the heterojunctions, 0.1 g of g-C_3_N_4_ and 0.045 g of BiOI NSs were separately weighed. Subsequently, they were mixed with anhydrous ethanol and ground together until the ethanol evaporated completely. The resulting orange product was then transferred into a crucible and heated in a muffle furnace at a rate of 5 °C per minute up to 300 °C. The sample was calcined for 2 h and then cooled to obtain the g-C_3_N_4_/BiOI nanocomposites. The detailed synthesis procedure is described in Scheme 1. Different amounts of BiOI photocatalysts were added during the reaction process, and the corresponding mass ratios are detailed in Table 2.

### 3.3. Characterization of Materials

X-ray diffraction (XRD) patterns were recorded using a Bruker D2 Phaser X-ray diffractometer, manufactured by Bruker AXS GmbH, located in Karlsruhe, Germany. The system was operated at 30 kV with Cu Kα radiation (λ = 1.5406 Å), a current of 10 mA, and a step size of 0.02°, scanning in the range of 5 to 80 degrees. The software version used for data acquisition was DIFFRAC.MEASUREMENT CENTER V7. X-ray photoelectron spectroscopy (XPS) was performed with a Thermo Fisher Scientific ESCALAB 250Xi system, manufactured by Thermo Fisher Scientific Inc., located in Waltham, MA, USA. The morphological characteristics of the prepared nanomaterials were analyzed using a Tecnai F20 transmission electron microscope (TEM) operated at 200 kV and a JSM-6701F scanning electron microscope (SEM) operated at 10 kV. UV-Vis diffuse reflectance spectra (UV-Vis DRS) in the range of 200–800 nm and UV-Vis absorption spectra in the range of 200–700 cm^−1^ were collected using a SHIMADZU UV-3600 Plus spectrophotometer, manufactured by Shimadzu Scientific Instruments (SSI), headquartered in Columbia, MD, USA. Surface area and pore size distribution of the samples were measured using a JW-BK200C analyzer (Beijing JWGB Sci & Tech Co., Ltd, Beijing, China) under a nitrogen atmosphere. Fourier transform infrared (FT-IR) spectra were obtained using a SHIMADZU IRTracer-100 spectrophotometer, manufactured by Shimadzu Scientific Instruments (SSI), headquartered in Columbia, MD, USA. Samples were thoroughly mixed with KBr and pressed into pellets. Photoluminescence (PL) spectra were acquired using a Hitachi F-4700 fluorescence spectrophotometer at room temperature with an excitation wavelength of 325 nm. In this electrochemical impedance spectroscopy (EIS) experiment, the potential was set at 0.1 V with an amplitude of 0.005 V using a 0.5 M Na_2_SO_4_ solution across a frequency range of 0.01 to 10 kHz.

### 3.4. Photocatalytic Degradation Experiment

During the photocatalytic process, the absorbance (*A*) of the analyte at different time intervals was measured using the UV-3600 Plus spectrophotometer. According to Lambert–Beer’s law, the *A* was proportional to the concentration of the target contaminant (*C*). The photocatalytic degradation efficiency (*D*) was calculated by Equation (5) as follows [74]:

(5) $$D=\frac{C_{0}-C_{t}}{C_{0}}\times 100\%=\frac{A_{0}-A_{t}}{A_{0}}\times 100\%$$

where *C*_0_ denotes the initial concentration of the target pollutant (mg/L), *C_t_* indicates the concentration of the target pollutant at time *t* during the photocatalytic process (mg/L), *A*_0_ stands for the initial absorbance values of the target pollutant, and *A_t_* stands for its absorbance values at time *t* during the photocatalytic process. The specific operation of the photocatalytic degradation experiment is as follows: using a Microsolar 300 photocatalytic device (Beijing Perfectlight Technology Co., Ltd., Beijing, China) as the reaction device to simulate sunlight, 10 mg of photocatalyst was poured into a beaker containing 10 mg/L of dye solution. To exclude the influence of the photocatalytic degradation efficiency by the adsorption of the catalyst and the effect of dye sensitization, the dye–photocatalyst mixture was placed in a dark room for 30 min to achieve adsorption–desorption equilibrium. Subsequently, the power was turned on, and the beaker was positioned 15 cm away from the light source. The variation in the Rh B absorption peak at 553 nm was recorded at 10-minute intervals to determine the contaminant concentrations during photocatalytic degradation. The photocatalytic time and photocatalytic conditions of the repeatability experiment were not changed. After each photocatalytic cycle, the photocatalyst was ultrasonically washed several times with absolute ethanol. Since the catalyst may be reduced during each wash, an analytical balance was used to weigh the photocatalyst and measure the degradation solution in the same ratio before proceeding to the next photocatalytic test. The operation was repeated 5 times to complete the repeatability test.

## 4. Conclusions

To summarize, we successfully prepared mesoporous 2D/2D g-C_3_N_4_/BiOI p-n heterojunctions through a combination of the precipitation method and high-temperature calcination. Compared to pure g-C_3_N_4_ NSs and BiOI NSs, the g-C_3_N_4_/BiOI p-n heterojunctions exhibited lower electron–hole pair recombination rates, increased photogenerated electrons, and smaller charge transfer resistances, resulting in significantly higher photocatalytic activity. Under simulated sunlight, the g-C_3_N_4_/BiOI heterojunction achieved a 99.7% degradation efficiency of Rh B within 60 min, representing an increase of 2.37 and 1.27 times over pure BiOI NSs and g-C_3_N_4_ NSs, respectively. The electron transfer mechanism in the g-C_3_N_4_/BiOI-45 p-n heterojunction followed a Z-mechanism, which was further validated by radical scavenging experiments. These experiments confirmed that ∙O_2_^−^ and ∙OH are the main reactive species responsible for the degradation process. Additionally, the degradation efficiency of the g-C_3_N_4_/BiOI-45 heterojunction decreased by only 1.2% after five cycles, and the XRD diffraction peaks remained unchanged, demonstrating the material’s excellent recyclability and stability. This study not only demonstrates the enhanced photocatalytic potential of 2D/2D heterojunctions but also provides an effective and low-cost method for their large-scale production, making them promising candidates for addressing environmental pollution issues.

## Figures and Tables

**Table 1:** List of diverse photocatalysts studied for degrading Rh B.

Sample	Amount	Application	Concentration and Usage	Power Source	Time	Efficiency	Ref.
Bi_4_Ti_3_O_12_/AgI heterojunction	20 mg	Rh B degradation	20 mg/L 60 mL	_	60 min	92.5%	Liu et al. [60]
Ag_2_CrO_4_/g-C_3_N_4_heterojunction	50 mg	Rh B degradation	2 × 10^−5^ M 100 mL	Xe lamp 300 W	120 min	94%	Alsalme et al. [61]
Bi_2_MoO_6_/Bi_2_Mo_3_O_12_ heterojunction	30 mg	Rh B degradation	10 mg/L 30 mL	Xe lamp 300 W	30 min	95%	Wang et al. [62]
BiVO_4_/g-C_3_N_4_ heterojunction	180 mg	Rh B degradation	10 mg/L 180 mL	Xe lamp 500 W	75 min	98%	Khan et al. [63]
Cu_2_O/BiOBr heterojunction	50 mg	Rh B degradation	100 mg/L 100 mL	Xe lamp 300 W	50 min	82.33%	Gao et al. [64]
β-Bi_2_O_3_/TiO_2_ heterojunction	50 mg	Rh B degradation	20 mg/L 60 mL	Xe lamp 300 W	60 min	99.6%	Wang et al. [65]
MoS_2_/ZnO heterojunction	25 mg	Rh B degradation	10 mg/L 50 mL	Xe lamp 200 W	50 min	95%	Gang et al. [66]
ZnIn_2_S_4_/Bi_2_WO_6_ heterojunction	10 mg	Rh B degradation	10 mg/L 50 mL	Xe lamp 300 W	40 min	93.49	Liu et al. [67]
BiOI/AgEuW_2_O_8_ heterojunction	60 mg	Rh B degradation	30 mg/L 100 mL	Xe lamp 300 W	180 min	91.77%	Wu et al. [68]
CuO/MoO_3_ heterojunction	15 mg	Rh B degradation	8 mg/L 60 mL	Xe lamp 400 W	120 min	99%	Hussain et al. [69]
BiOBr/CdS heterojunction	20 mg	Rh B degradation	10 mg/L 40 mL	LED light 5 W	60 min	97%	Yu et al. [70]
SnO_2_/g-C_3_N_4_ composite	100 mg	Rh B degradation	30 mg/L 200 mL	LED light 30 W	150 min	99.42%	Van et al. [71]
CuWO_4_/CuS heterojunction	40 mg	Rh B degradation	15 mg/L 100 mL	Xe lamp 300 W	90 min	93.2%	Cui et al. [72]
CuO/g-C_3_N_4_ heterojunction	100 mg	Rh B degradation	5 mg/L 100 mL	Xe lamp 300 W	30 min	88.9%	Zhu et al. [73]

**Table 2:** Composition ratios of g-C_3_N_4_ and BiOI and the corresponding nomenclature for g-C_3_N_4_/BiOI samples.

g-C_3_N_4_ (mg)	BiOI (mg)	Symbols of g-C_3_N_4_/BiOI
100	25	g-C_3_N_4_/BiOI-25
100	35	g-C_3_N_4_/BiOI-35
100	45	g-C_3_N_4_/BiOI-45
100	55	g-C_3_N_4_/BiOI-55
100	65	g-C_3_N_4_/BiOI-65

## Data Availability

Data are contained within the article.
